# Electric Properties of Multiwalled Carbon Nanotubes Dispersed in Liquid Crystals and Their Influence on Freedericksz Transitions

**DOI:** 10.3390/nano12071119

**Published:** 2022-03-28

**Authors:** Emil Petrescu, Cristina Cirtoaje

**Affiliations:** Department of Physics, Faculty of Applied Science, University Politehnica of Bucharest, RO-060042 Bucharest, Romania; emil.petrescu@upb.ro

**Keywords:** carbon nanotubes, nematic liquid crystals, Freedericksz transition, electric properties

## Abstract

Liquid crystal composites with multiwalled carbon nanotubes present dielectric properties considerably different from those of pure liquid crystal (LC). Using a proper dispersion of nanotubes in the LC-sample and a theoretical model in agreement with the experimental configuration, the dielectric permittivities of multiwalled carbon nanotubes are calculated. The influence of dielectric properties on the Freedericksz transition threshold is discussed. Theoretical values for dielectric permittivities of multiwalled carbon nanotubes are calculated for different temperatures

## 1. Introduction

Recently, an increased interest of scientists and engineers on nano- and micro-materials led to a fast development of new materials and devices. Any materials containing particles with less than 100 nm in diameter are considered nanomaterials and can be used as thin films or surface coatings, on computer chips, as nanowires, nanotubes, or as liquid dispersion of tiny nanocrystalline particles. In the beginning, nanoparticles were observed as naturally occurring elements in volcanic ash, as residual elements, or as emissions in the combustion process of power plant chimneys and diesel engines, and they were known as ultrafine particles. After the development of the scanning tunneling microscope, nanomaterials were deliberately synthesized in different laboratories to improve the material’s physical and chemical properties for a specific purpose or function. Carbon nanotubes (CNT) are some of the first discovered and synthesized nanomaterials [1,2,3,4,5]. They are also the most studied and used in various fields, from electronic and communication engineering [6,7,8] to chemistry [9,10], environmental science [11,12] and medicine [13,14]. Initially, experimental and theoretical studies were made on their physical properties such as magnetic properties [15,16,17,18], optical transmission and absorbency [19,20], electrical or thermal conductivity [21,22,23] and mechanical strength or elasticity [24]. More advanced theoretical models were proposed to provide a deeper understanding of phenomena in the nanoscale world including nanoparticles and/or liquid crystal molecules [25,26,27,28,29,30,31]. Although the applications might be various and probably will bring a considerable improvement to many systems, some suspicion appears as a result of incomplete knowledge of their physical properties [12], and information about toxic effects on devices functionality or even on life itself, which may be induced by ignorance. A serious problem when trying to study CNT’s properties comes from organization. Isotropic powders or dispersion present randomly oriented nanotube axes and, thus, some properties may be strongly affected, leading to a behavior more similar to macroscopic bulk than nanoparticles ensemble. Without a proper alignment, especially for long particles such as nanotubes or nanowires, their true potential cannot be reached. A soft organized environment is needed. Liquid crystals are good candidates because they seem to align their director with the nanotube’s long axis [32,33,34,35]. Starting from this point, we designed a theoretical model to describe the electro-optic behavior of carbon nanotubes dispersion in nematic liquid crystals at room temperature. The dielectric permittivities of carbon nanotubes were evaluated and their influence on Freedericksz transition threshold voltage of 7CB composite with multiwalled carbon nanotubes (MWCNT) was discussed.

## 2. Theory

**Figure 1:**
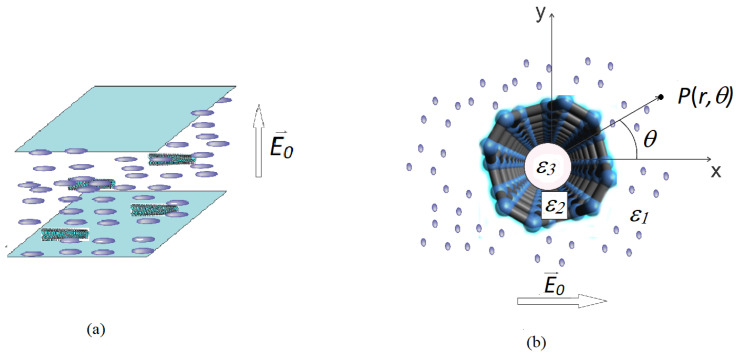
Planar cell containing LC+MWCNT composite: (**a**) the whole cell view, (**b**) inside view.

**Figure 2:**
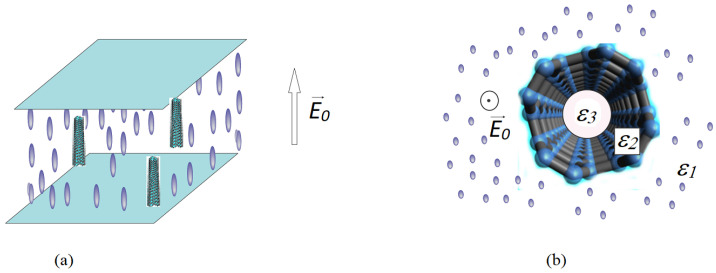
Homeotropic cell containing LC + MWCNT composite. (**a**) the whole cell view, (**b**) inside view.

When a low concentration of CNTs are inserted into a liquid crystal cell, the molecules tend to align themselves parallel to the nanotube’s long axis as represented in Figure 1 and Figure 2. This alignment influences their orientation under an external field, and thus, the Freedericksz transition threshold voltage.

As presented in [36] the threshold voltage can be evaluated by:

(1) $$U=U_{0}\sqrt{\frac{\varepsilon_{a}}{\varepsilon_{a\;eff}}\left(1-\frac{4wfL^{2}}{\pi^{2}K_{1}a}\right)}$$

There are several factors that can affect the transition. One of them is the temperature which affects the Frank elastic constant $K_{1}$ and induces a decrease in the threshold voltage on a specific range where the order is reasonably maintained [37,38]:

(2) $$K_{i}=K_{i0}{\left(1-\frac{T}{T_{c}}\right)}^{2\beta }$$

The coefficient *i* refers to the order of the elastic constant (*i* = 1, 2, 3 for splay, twist and bend elastic deformation), *T* is the absolute temperature of the sample, $T_{c}$ is the clearing temperature of the liquid crystal, β is a material constant [37] and $K_{i0}$ are the elastic constants at T= 273.15 K.

Another factor, is the anchoring energy density (w) through the interaction process described by Burylov and Zakhlevnykh in [30]:

(3) $$F\left(w,f\right)=\frac{4wfL^{2}}{\pi^{2}K_{1}a}$$

where *w* is the anchoring energy density, *f* is the volumetric fraction of nanotubes, *L* is the cell’s thickness, $K_{1}$ is the splay elastic constant and *a* is the nanotube outer radius.

Finally there is the dielectric anisotropy of the composite $\varepsilon_{a\;eff}$ which can be controlled by the amount of carbon nanotubes inserted into the sample. This can be performed with a precise knowledge of the nanotube’s permittivity and of the microscopic processes involved in the sample’s configuration. For the carbon nanotube’s dispersion in planar aligned cell, (Figure 1) we consider a physical system of an empty semiconductor cylinder in a dielectric environment. This system is in agreement with the experiment because, the liquid crystal, which is quite viscous, will not enter inside the narrow tube. If an external electric field $E_{0}$ is applied to the LC cell, its direction will be perpendicular to the molecular direction and to the nanotube’s axis as shown in Figure 1b. Due to the polarization effects, the effective field inside sample (E), can be calculated as an average field on a large domain of the mixture. According to this domain, the mixture behaves like a homogeneous environment described by electric displacement *D*. By integrating the entire cell volume we obtain:

(4) $$\frac{1}{V}\int_{V}\left(D-\varepsilon_{1}\varepsilon_{0}E\right)dv=\bar{D}-\varepsilon_{1}\varepsilon_{0}\bar{E}$$

where *V* is the mixture’s volume, $\varepsilon_{0}$ is the vacuum electric permittivity and $\varepsilon_{1}$ is the dielectric constant of LC. $\bar{E}$ and $\bar{D}$ are the average field parameters in the mixture. The integral covers a succession of three regions with different permittivities as shown in Figure 1b: the first one is the air inside the tube with the dielectric permittivity $\varepsilon_{0}$, the second one is the nanotube’s wall with the permittivity $\varepsilon_{2}$ and the third one is the liquid crystal surrounding the tube with the permittivity $\varepsilon_{1}$ so Equation (4) becomes:

(5) $$\begin{array}{c} \frac{1}{V}\int_{ext}\left(D_{1}-\varepsilon_{1}\varepsilon_{0}E_{1}\right)dv+\frac{N}{V}\int_{0}^{b}\left(D_{3}-\varepsilon_{1}\varepsilon_{0}E_{3}\right)2\pi rldr+\\ \frac{N}{V}\int_{b}^{a}\left(D_{2}-\varepsilon_{1}\varepsilon_{0}E_{2}\right)2\pi rldr=\bar{D}-\varepsilon_{1}\varepsilon_{0}\bar{E} \end{array}$$

where *l* is the nanotube length and *N* is the nanotubes number in the mixture, $D_{1}=\varepsilon_{1}\varepsilon_{0}E_{1}$ is the electric displacement around CNT in LC, $D_{2}=\varepsilon_{2}\varepsilon_{0}E_{2}$ is the electric displacement inside the nanotube wall and $D_{3}=\varepsilon_{3}\varepsilon_{0}E_{3}=\varepsilon_{0}E_{3}$ is the electric displacement inside the tube (Figure 1), *a* is the outer radius of the tube and *b* is its inner radius.

It can be easily observed that the first integral is null because $D_{1}=\varepsilon_{1}\varepsilon_{0}E_{1}$, so we only have two integrals:

(6) $$I_{2}=\int_{b}^{a}\left(D_{2}-\varepsilon_{1}\varepsilon_{0}E_{2}\right)2\pi rldr$$

(7) $$I_{3}=\int_{0}^{b}\left(D_{3}-\varepsilon_{1}\varepsilon_{0}E_{3}\right)2\pi rldr$$

Using the electrostatic principles of the dielectrics described by Landau and Lifsit in [39], we can calculate the integral using the electric field in each region from the electric potential of the applied field in a point P determined by cylindrical coordinates *r* and θ. For a point situated outside the nanotube the potential is:

(8) $$\varphi_{1}=-E_{0}\cos\theta \left(r-\frac{A}{r}\right)\;r>a,$$

for a point situated inside the nanotube’s wall we obtain:

(9) $$\varphi_{2}=-CE_{0}\cos\theta \left(r-\frac{G}{r}\right)\;b\leq r\leq a$$

and for a point inside the CNT it results:

(10) $$\varphi_{3}=-E_{0}rB\cos\theta$$

where *A*, *B*, *C* and *G* are constants and can be determined from the boundary conditions of each region from which we obtain:

(11) $$\begin{array}{c} \varphi_{1}\left(a\right)=\varphi_{2}\left(a\right)\\ \varphi_{2}\left(b\right)=\varphi_{3}\left(b\right)\\ \varepsilon_{1}\varepsilon_{0}{\left.\frac{\partial \varphi_{1}}{\partial r}\right|}_{r=a}=\varepsilon_{2}\varepsilon_{0}{\left.\frac{\partial \varphi_{2}}{\partial r}\right|}_{r=a}\\ \varepsilon_{2}\varepsilon_{0}{\left.\frac{\partial \varphi_{1}}{\partial r}\right|}_{r=b}=\varepsilon_{0}{\left.\frac{\partial \varphi_{3}}{\partial r}\right|}_{r=b} \end{array}$$

Finally it results:

(12) $$B=\frac{2}{\left(1+\frac{\varepsilon_{2}}{\varepsilon_{1}}\right)-\frac{\varepsilon_{2}-1}{\varepsilon_{2}+1}\left(\frac{\varepsilon_{2}}{\varepsilon_{1}}-1\right)\frac{b^{2}}{a^{2}}}\left(1+\frac{\varepsilon_{2}-1}{\varepsilon_{2}+1}\right)$$

(13) $$G=-\frac{\left(\varepsilon_{2}-1\right)}{\left(\varepsilon_{2}+1\right)}b^{2}$$

(14) $$C=\frac{2}{\left(1+\frac{\varepsilon_{2}}{\varepsilon_{1}}\right)-\frac{\varepsilon_{2}-1}{\varepsilon_{2}+1}\left(\frac{\varepsilon_{2}}{\varepsilon_{1}}-1\right)\frac{b^{2}}{a^{2}}}$$

Considering the external field $E_{0}$ parallel to Ox axis, we have x=rcosθ and we obtain:

(15) $$E_{2}=-\frac{\partial \varphi_{2}}{\partial x}=CE_{0}\left[1+\frac{G}{r^{2}}\right]$$

and

(16) $$E_{3}=BE_{0}$$

Using Equations (15) and (16) in Equations (6) and (7) we obtain:

(17) $$I_{2}=\pi a^{2}l\varepsilon_{0}I_{02}E_{0}$$

(18) $$I_{3}=\pi a^{2}l\varepsilon_{0}I_{03}E_{0}$$

where

(19) $${I_{0}}_{2}=\frac{2(\varepsilon_{2}-\varepsilon_{1})}{\left(1+\frac{\varepsilon_{2}}{\varepsilon_{1}}\right)-\frac{\varepsilon_{2}-1}{\varepsilon_{2}+1}\left(\frac{\varepsilon_{2}}{\varepsilon_{1}}-1\right)\frac{b^{2}}{a^{2}}}\left[\left(1-\frac{b^{2}}{a^{2}}\right)-\frac{\varepsilon_{2}-1}{\varepsilon_{2}+1}\frac{b^{2}}{a^{2}}\ln\frac{a}{b}\right]$$

(20) $${I_{0}}_{3}=\frac{2(1-\varepsilon_{1})}{\left(1+\frac{\varepsilon_{2}}{\varepsilon_{1}}\right)-\frac{\varepsilon_{2}-1}{\varepsilon_{2}+1}\left(\frac{\varepsilon_{2}}{\varepsilon_{1}}-1\right)\frac{b^{2}}{a^{2}}}\left(1+\frac{\varepsilon_{2}-1}{\varepsilon_{2}+1}\right)\frac{b^{2}}{a^{2}}$$

By denoting $v=\pi a^{2}l$ the volume of a single nanotube, Equation (5) becomes:

(21) $$\frac{Nv}{V}\varepsilon_{0}E_{0}\left[I_{02}+I_{03}\right]=\bar{D}-\varepsilon_{1}\varepsilon_{0}\bar{E}$$

Considering the applied field $E_{0}$ is equal to the average field inside the mixture $\bar{E}$ and replacing $\bar{D}=\varepsilon_{eff}\varepsilon_{0}\bar{E}$, we obtain:

(22) $$\varepsilon_{eff}=\varepsilon_{1}+f\left(I_{02}+I_{03}\right)$$

where $f=\frac{Nv}{V}$ is the volumetric fraction of carbon nanotubes in the mixture.

In this case, the field is perpendicular to the nanotube axis, and the nanotubes are parallel to the molecular director, so $\varepsilon_{1}\equiv \varepsilon_{⊥LC}$ and $\varepsilon_{2}\equiv \varepsilon_{⊥CNT}$. Thus, we obtain:

(23) $$\begin{array}{c} \varepsilon_{⊥eff}=\varepsilon_{⊥LC}+\frac{2f(\varepsilon_{2}-\varepsilon_{1})}{\left(1+\frac{\varepsilon_{2}}{\varepsilon_{1}}\right)-\frac{\varepsilon_{2}-1}{\varepsilon_{2}+1}\left(\frac{\varepsilon_{2}}{\varepsilon_{1}}-1\right)\frac{b^{2}}{a^{2}}}\left[\left(1-\frac{b^{2}}{a^{2}}\right)-\frac{\varepsilon_{2}-1}{\varepsilon_{2}+1}\frac{b^{2}}{a^{2}}\ln\frac{a}{b}\right]\\ +\frac{2f(1-\varepsilon_{1})}{\left(1+\frac{\varepsilon_{2}}{\varepsilon_{1}}\right)-\frac{\varepsilon_{2}-1}{\varepsilon_{2}+1}\left(\frac{\varepsilon_{2}}{\varepsilon_{1}}-1\right)\frac{b^{2}}{a^{2}}}\left(1+\frac{\varepsilon_{2}-1}{\varepsilon_{2}+1}\right)\frac{b^{2}}{a^{2}} \end{array}$$

In expressions of $I_{02}$ and $I_{03}$ we also have $\varepsilon_{1}\equiv \varepsilon_{⊥LC}$ and $\varepsilon_{2}\equiv \varepsilon_{⊥CNT}$.

The parallel component of the mixture’s electric permittivity can be calculated in a similar way considering a homeotropic cell where the field director is parallel to the nanotube’s axis as it is shown in Figure 1. In this case, we obtained:

(24) $$\varepsilon_{\|\;eff}=\varepsilon_{\|LC}\left\{1+f\left[\left(1-\frac{\varepsilon_{\|CNT}}{\varepsilon_{\|LC}}\right)\frac{b^{2}-a^{2}}{a^{2}}+\left(1-\varepsilon_{\|LC}\right)\frac{b^{2}}{a^{2}}\right]\right\}$$

Using Equations (23) and (24) we can evaluate the dielectric anisotropy:

(25) $$\varepsilon_{a\;eff}=\varepsilon_{\|\;eff}-\varepsilon_{⊥eff}$$

Equation (25) offers the possibility to evaluate the dielectric permittivities of carbon nanotubes and allows a control of the Freedericksz transition threshold.

## 3. Materials and Methods

Previous research on carbon nanotubes dispersed in liquid crystals, reported a decrease in transition threshold when carbon nanotubes were added. In order to reproduce this effect and obtain a consistent decrease in the transition voltage, we prepared two samples with higher concentrations to be used in experimental research [40,41,42]. The analyzed samples were prepared by mixing 7CB nematic with multiwalled carbon nanotubes with the average length of 10 μm inner diameter of 2b= 4.5 nm and the outer diameter 2a=10 nm. The nanotubes were dispersed in toluene and sonicated for several hours until a good dispersion was obtained. The solution was mixed with the nematic and placed in a laboratory clean room to evaporate the toluene. The mixture was weighed daily until the weight was constant and the evaporation process was finished. The resulted nanotubes concentrations were 0.36 wt% and 1.30 wt%. These mixtures were used to fill 15 μm thick planar aligned cell from Instek and 15 μm homeotrpic cells prepared by us. Both types of cells have the effective area of 1 cm${}^{2}$. The Franck constants of 7CB were considered as given in [43] to be $K_{1}=6.716\times 10^{-12}$ N and $K_{2}=3.674\times 10^{-12}$ N.

**Figure 3:**
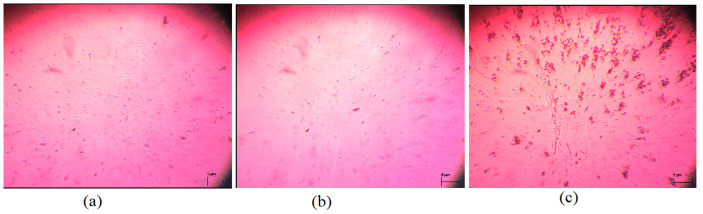
Polarized microscopy images recorded for planar aligned cells: (**a**) for 7CB sample, (**b**) for 7CB + 0.36% MWCNT and (**c**) for 7CB + 1.30% MWCNT.

The samples were analyzed by a polarized light microscope and it resulted that for high concentrations a clustering phenomena occurs leading to irregular micrometric structures as shown in Figure 3. The sample with a higher concentration of nanotubes presented an increase in the transition voltage due to agglomerations and due to different anchoring processes on microparticles formed inside the sample. For this structure of micrometric bulk dispersion, we cannot use the theoretical models presented in the previous section. For the lower concentration sample, the image is similar to the one obtained for the pure 7CB so the molecular order is maintained and the nanotubes are well dispersed and aligned parallel to the nematic director as considered in the theory. For the 0.36% mass fraction of MWCNT with the nanotube powder concentration provided by the supplier (ρ = 0.07 g/cm${}^{3}$), we obtained a volumetric fraction f=6.5%. In this case, we obtained a decrease in the Freedericksz transition as indicated in [40,41,42].

**Figure 4:**
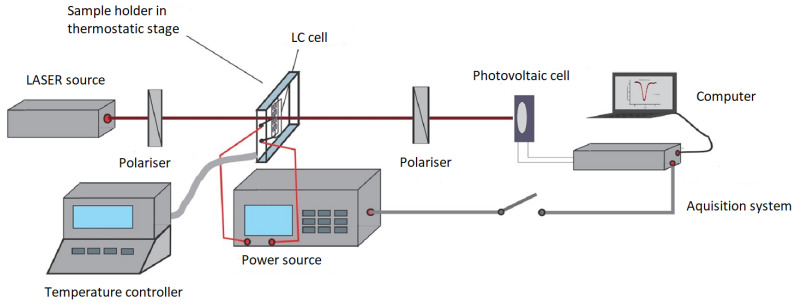
Experimental set-up for Freedericksz transition threshold.

The measurements were performed on the set-up presented in Figure 4. A laser beam from the source was sent through the sample placed between two crossed polarizers. The polarizer’s axes were set at 45${}^{\circ }$ to the laser beam polarization direction to ensure the equal intensity for both ordinary and extraordinary rays. The emergent beam was recorded by a photovoltaic cell and the signal was sent to the computer. The power source used in the set-up has two functions: it can be used to apply a precise voltage on the sample at a set frequency but it can also record the capacity of the cell.

**Figure 5:**
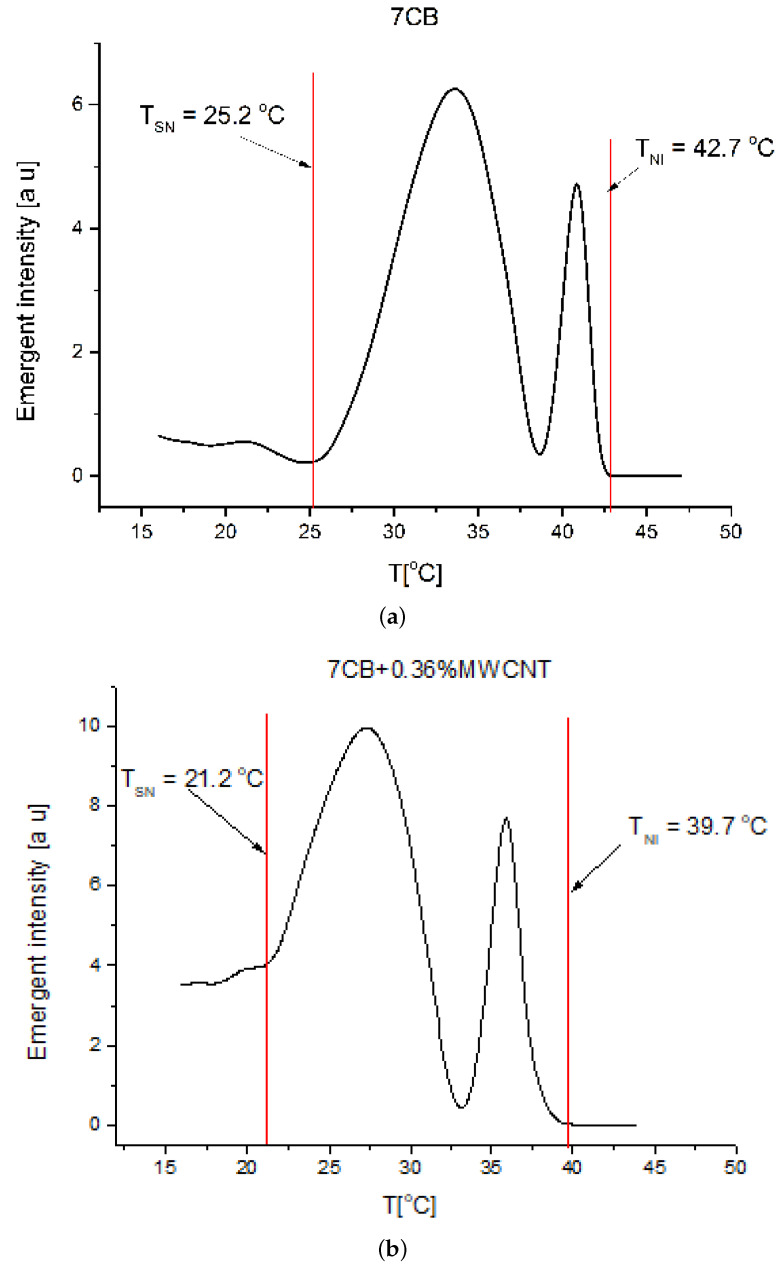
Emergent intensity versus temperature (**a**) for 7CB and (**b**) for 7CB + 0.36% MWCNT.

**Figure 6:**
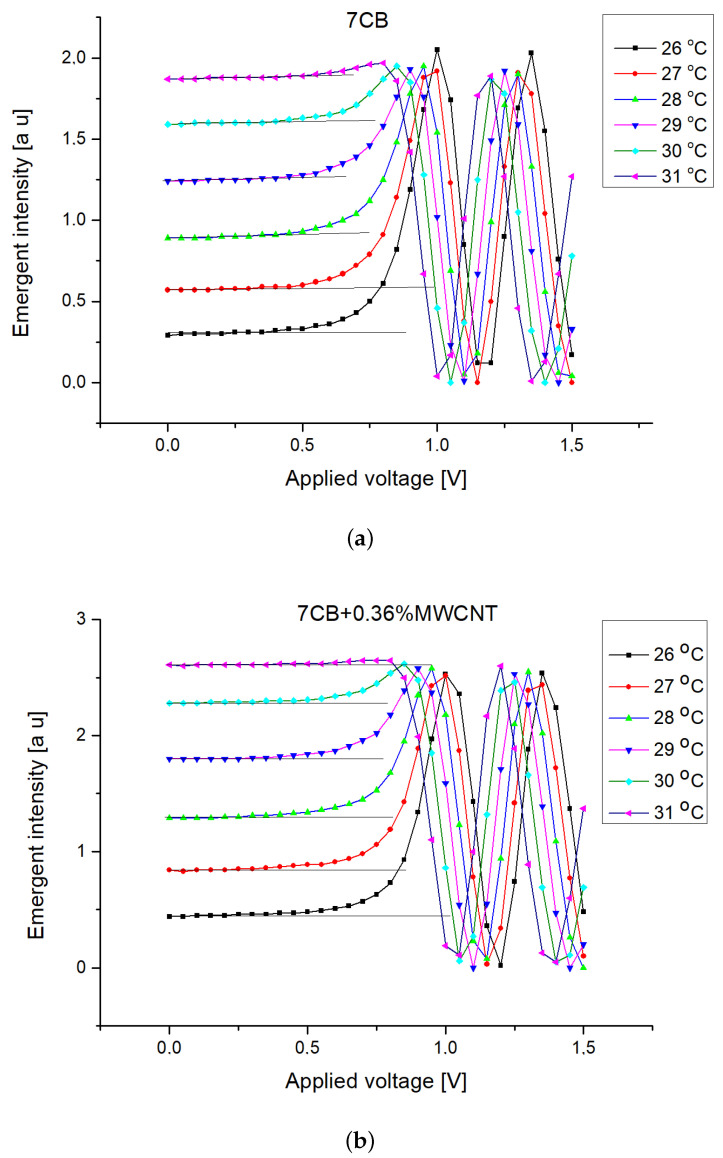
Emergent intensity versus applied voltage plots (**a**) for 7CB and (**b**) for 7CB + 0.36% MWCNT.

The nematic range was evaluated from the intensity versus temperature plot for each sample. The liquid crystal phase corresponds to the domain where significant intensity variation occurs due to the molecular reorientation induced by the laser beam (Figure 5). For the Freedericksz transition evaluation the capacity acquisition was switched off and the electric field applied was slowly increased by applying an alternate voltage (10 kHz) from the power source The emergent beam intensity was determined for each voltage after a resting time of 2 s (much longer than the relaxation time of the mixtures to be sure that the systems is stabilized). The intensity versus applied voltage plots were recorded and the Freedericksz transition threshold was experimentally determined as the point where intensity starts the increase (Figure 6).

**Figure 7:**
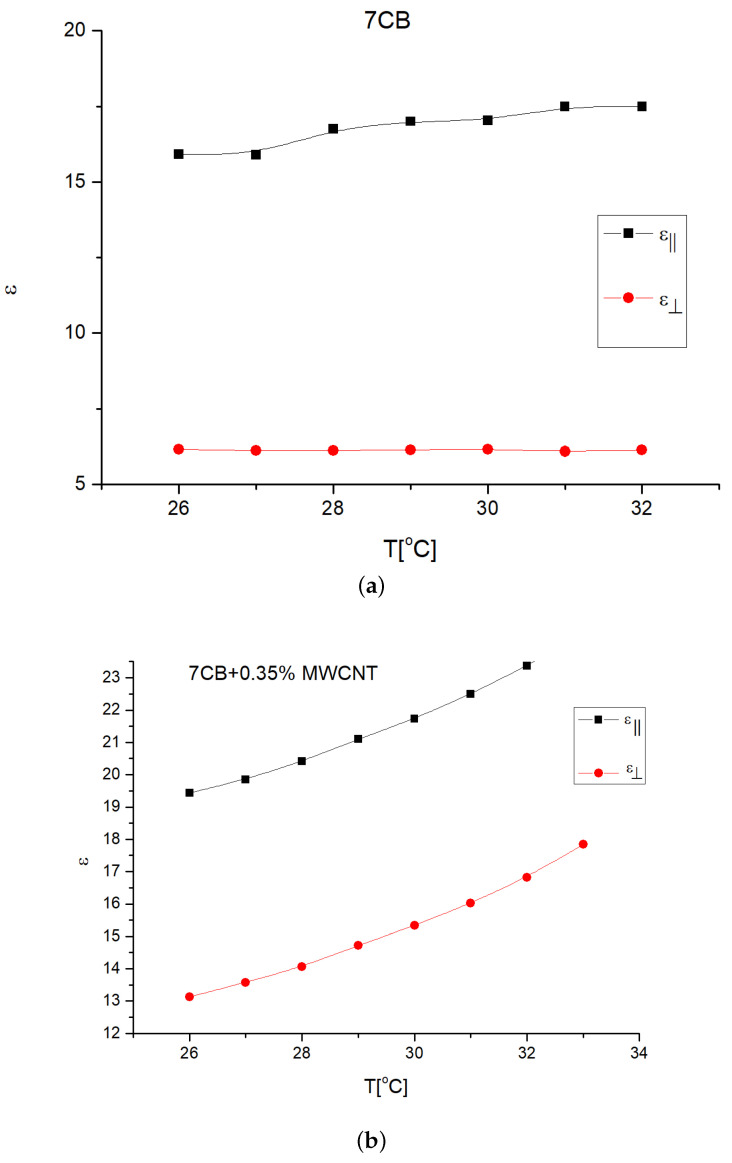
Parallel and perpendicular permittivities for (**a**) 7CB and (**b**) 7CB + 0.36% MWCNT.

For the capacity measurements, the laser source was switched off and the the acquisition system was enabled. The parallel capacitance was determined for the homeotropic cell and the perpendicular capacity was measured in the homogeneous aligned cell. The obtained values were adjusted by the capacitances of the empty cell.The dielectric permittivities were calculated from the capacitances by multiplying them with the thickness/active area ratio (the active area of each sample is 1 cm${}^{2}$ and the thickness is 15 microns). The results are presented in (Figure 7). For the temperature depending plots, the LC cell was placed in the hot stage of a Mettler-Toledo thermo-stabilized heat source in which the temperature can be set by a precision of 0.01 degrees Celsius.

## 4. Results and Discussion

The Freedericksz transition voltage evaluated from Intensity versus Voltage plots given in Figure 6, and the dielectric anisotropy calculated from the permittivities given in Figure 7 are presented in Table 1. The temperature range was chosen in the first part of the nematic range of 7CB to keep the elastic constant variations very small around the average values of $K_{1}=6.716\times 10^{-12}$ N and $K_{2}=36.74\times 10^{-12}$ N in agreement with Equation (2) with a material constant of β=0.1855.

As it can be observed, there is a considerable decrease (about 30%) in the threshold value for the sample containing multiwalled carbon nanotubes compared to the reference sample containing LC only. According to the formula given in Equation (1), this decrease may be explained by an increase in the effective anisotropy of the sample or by a variation of the anchoring energy of the molecules on the nanotube’s surface. If we rewrite Equation (1) in a different form, we can use:

(26) $$\frac{U}{U_{0}}=\sqrt{\frac{\varepsilon_{a}}{\varepsilon_{a\;eff}}}\sqrt{1-F(w,f)}$$

where $F\left(w,f\right)=\frac{4wfL^{2}}{\pi^{2}K_{1}a}$ is the anchoring depending term.

As it can be observed from Equation (26), we can decrease the threshold voltage *U* by increasing the anchoring energy or by increasing the nanotube’s anisotropy.

For our specific materials, the value of F(w,t) presented in Table 1 varies from 0.39 to 0.44 depending on the temperature but it can be adjusted by chemical functionalization of the nanotubes or by other chemical processes. A detailed discussion about this energy is provided in [35,36,39]. An increase in MWCNT concentration in the sample may also reduce the transition threshold but it must be performed with caution because it may induce the clustering effect as can be seen from Figure 3. Another way to decrease the threshold voltage is to increase the dielectric anisotropy by using other types of carbon nanotubes with different lengths and thicknesses.

Using Equations (23)–(25), we can calculate the parallel dielectric permittivity ($\varepsilon_{\|}$), the perpendicular permittivity ($\varepsilon_{⊥}$) and the dielectric anisotropy of carbon nanotubes ($\varepsilon_{aCNT}$). For the nanoparticles used in this experiment, the obtained permittivities are given in Table 2.

It can be observed from the data obtained in Table 2 that MWCNT present a considerable higher dielectric anisotropy compared to the liquid crystals, and it may explain the composite reorientation at lower voltages and the decrease in the Freedericksz transition. It is also important to mention that, if the geometrical parameters of the nanotubes are known, the dielectric permittivities can be calculated if the effective anisotropy is known. Electro-optic applications, such as ultra-broadband electromagnetic wave absorption, Ref. [44] can benefit from the evaluation of parameters. An interesting aspect here is the temperature dependence of dielectric anisotropy. For the 7CB nematic, it increases with the temperature, while for nanotubes, we can notice it strongly decreases. Due to the small volumetric fraction of impurity the overall behavior is similar to the one of the liquid crystal but for the proper control of the transition threshold one must consider both dependencies.

## 5. Conclusions

This manuscript presents a discussion about the ways in which the carbon nanotubes inserted in the liquid crystal matrix can affect its dielectric properties. The nanotube content affects the average dielectric anisotropy and it can affect the temperature effect on Freedericksz transition. There are two possible applications of the aspects discussed in this article. The first one is from an engineering point of view: it can be used for the decrease in the transition threshold voltage by adjusting the amount of nanotubes or by using different coatings that may influence the anchoring strength or angle. This can be an advantage for LCDs because a lower transition voltage leads to a lower power consumption and a longer life for the batteries. From a scientific point of view, we proposed a theoretical method to determine the dielectric parallel and permittivity of carbon nanotubes. The liquid crystal environment is a proper tool to obtain an organized nanotubes ensemble as considered in the discussed theory. Thus, by simple measurements of LC cell capacity when filled with an LC+CNT composite, one can calculate the nanotube’s permittivities.

## Figures and Tables

**Table 1:** Electric Freedericksz transition threshold for 7CB ($U_{0}$) and for 7CB + MWCNTs (U).

*T* (${}^{\circ }$C)	$U_{0}\left[V\right]$	U[V]	$\varepsilon_{a}$	$\varepsilon_{\mathrm{aeff}}$	$U_{0}/U$	$\sqrt{\varepsilon_{a}\varepsilon_{\mathrm{aeff}}}$	F(w,f)
26	0.37	0.25	9.76	13.14	0.68	0.86	0.39
27	0.36	0.23	9.78	13.57	0.64	0.85	0.43
28	0.41	0.26	10.63	14.06	0.63	0.87	0.47
29	0.43	0.27	10.87	14.72	0.63	0.86	0.47
30	0.43	0.28	10.87	15.25	0.65	0.84	0.41
31	0.41	0.26	11.41	15.96	0.63	0.85	0.44

**Table 2:** Dielectric permittivities and anisotropy for MWCNT.

*T* (${}^{\circ }$C)	$\varepsilon_{\|\mathrm{CNT}}$	$\varepsilon_{⊥\mathrm{CNT}}$	$\varepsilon_{a\mathrm{CNT}}$
26	42.66	15.11	27.55
27	44.84	16.21	28.67
28	45.85	20.45	25.46
29	48.36	21.30	27.09
30	50.98	30.16	20.77
31	53.53	41.30	12.23
32	57.18	49.37	7.81

## Data Availability

Not applicable.
